# mTOR regulation of metabolism limits LPS-induced monocyte inflammatory and procoagulant responses

**DOI:** 10.1038/s42003-022-03804-z

**Published:** 2022-08-26

**Authors:** Nina C. Lund, Yetunde Kayode, Melanie R. McReynolds, Deanna C. Clemmer, Hannah Hudson, Isabelle Clerc, Hee-Kyung Hong, Jason M. Brenchley, Joseph Bass, Richard T. D’Aquila, Harry E. Taylor

**Affiliations:** 1 Division of Infectious Diseases, Department of Medicine, Northwestern University Feinberg School of Medicine, Chicago, IL 60611 USA; 2 Department of Microbiology & Immunology, SUNY Upstate Medical University, Syracuse, NY 13210 USA; 3 Department of Biochemistry and Molecular Biology, Huck Institutes of the Life Sciences, Pennsylvania State University, University Park, PA 16802 USA; 4 Division of Endocrinology, Department of Medicine, Northwestern University Feinberg School of Medicine, Chicago, IL 60611 USA; 5 Barrier Immunity Section, Laboratory of Viral Disease, National Institute of Allergy and Infectious Diseases, Bethesda, MD 20892 USA

**Keywords:** HIV infections, Monocytes and macrophages

## Abstract

Translocated lipopolysaccharide (LPS) activates monocytes via TLR4 and is hypothesized to increase cardiovascular disease risk in persons living with HIV. We tested whether mTOR activity supports LPS-stimulated monocyte production of pro-inflammatory cytokines and tissue factor (TF), as it propels the inflammatory response in several immune cell types besides monocytes. However, multi-omics analyses here demonstrate that mTOR activates a metabolic pathway that limits abundance of these gene products in monocytes. Treatment of primary human monocytes with catalytic mTOR inhibitors (mTORi) increased LPS-induced polyfunctional responses, including production of IL-1β, IL-6, and the pro-coagulant, TF. NF-κB-driven transcriptional activity is enhanced with LPS stimulation after mTORi treatment to increase expression of *F3* (TF). Moreover, intracellular NAD^+^ availability is restricted due to decreased salvage pathway synthesis. These results document mTOR-mediated restraint of the LPS-induced transcriptional response in monocytes and a metabolic mechanism informing strategies to reverse enhanced risk of coagulopathy in pro-inflammatory states.

## Abstract (web summary)

mTOR limits the activation of an inflammatory gene response in monocytes. Inhibitors of mTOR enhance NF-κB-driven transcription in LPS-stimulated monocytes while depleting NAD^+^ through effects on the salvage pathway.

## Introduction

Persons living with HIV (PLWH) now face a growing burden of non-AIDS co-morbidities, rather than progressive immunodeficiency. Co-morbidities include a range of cardiovascular disease (CVD) pathologies^1–4^. Excess risk for PLWH is in part attributed to persistent immune activation resulting from microbial translocation across an impaired gut barrier^5–9^. Translocated microbial products, including lipopolysaccharide (LPS), activate innate immune cells, leading to the production of inflammatory mediators and hemostatic factors^10,11^. Therapeutic interventions addressing aberrant immune activation and coagulation could reverse these healthy lifespan-limiting comorbidities that may persist during viremia-suppressing antiretroviral therapy (ART).

Immune function relies on rapid responses to antigenic and inflammatory signals by highly specialized cells. During an immune response, naïve and memory T cells shift from a resting catabolic state and oxidative phosphorylation (OXPHOS) to growth, proliferation, and glycolysis—the “Warburg effect”—fueling the energetic demands, differentiation, proliferation, and cytokine production of these specialized cells^12,13^. At the center of this shift is the mechanistic target of rapamycin (mTOR), a conserved serine/threonine kinase that forms two complexes (mTORC1 and mTORC2) with distinct functions regulating metabolic pathways^14^.

The metabolic consequences of CD4^+^ T cell activation also dictate susceptibility to HIV. We previously established that mTOR activity governs increased susceptibility to HIV-1 infection after T cell activation by up-regulating biosynthesis of macromolecules required for HIV reverse transcription (RT) and cytoplasmic transport of RT products; along with others, we find that ATP-competitive, catalytic mTOR inhibitors (mTORi) targeting both enzymatic complexes efficiently suppress HIV replication, providing support for further assessing mTOR inhibitors as an adjunct to current ART^15–18^.

Signaling through toll-like receptors (TLRs) in myeloid-lineage cells also results in cell type-dependent activation of both mTOR complexes. LPS, which engages TLR4^19^, is found in blood at elevated concentrations in PLWH^6^. TLR4-activation of monocytes by LPS suppresses OXPHOS in favor of a glycolytic program that supports inflammatory cytokine production^6,20^. Congruently, compared to cells from uninfected subjects, monocytes obtained from virally suppressed PLWH show increased glucose transporter 1 (Glut1) expression and more readily differentiate to lipid-laden foam cells, attributed in part to mTOR-dependent accumulation of cellular cholesterol consequent to upregulated, receptor-mediated uptake of LDL^21–23^. Given the central role of mTOR in coordinating intracellular utilization of glucose and cholesterol, its inhibition could address aberrant monocyte activation, inflammatory cytokine production, and atherogenic activity. This hypothesis has already led to rapamycin, an allosteric mTORC1 inhibitor, being tested clinically as an adjunct to ART to evaluate potential benefits in mitigation of immune activation as well as viral control^24^.

However, there is laboratory evidence showing that inhibition of mTOR potentiates myeloid cell pro-inflammatory responses to LPS, raising the possibility of unintended exacerbation of inflammation with mTOR inhibition^25^. Here, we have characterized effects of mTORi in primary human monocytes stimulated with LPS ex vivo using flow cytometry, transcriptomics, and metabolomics. Results indicate that mTOR activity limits inflammatory and procoagulant responses to LPS in monocytes. mTORi treatment enhanced NF-κB-driven transcription and surface expression of the pro-coagulant tissue factor (TF) in LPS-stimulated monocytes, while concomitantly depleting NAD^+^ by impacting salvage pathway synthesis.

## Results

### mTORi pretreatment increased production of pro-inflammatory cytokines by primary human monocytes stimulated with LPS

**Fig. 1:**
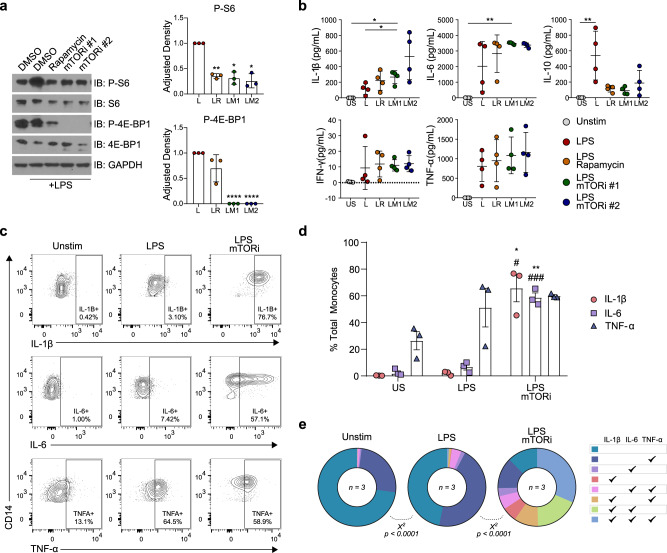
mTOR inhibition potentiates the inflammatory phenotype of primary uninfected human monocytes following TLR4 stimulation. **a** Freshly isolated human monocytes were pretreated as indicated with rapamycin (100 nM) or one of two structurally distinct mTORi (AZD2014, mTORi #1; INK128, mTORi #2; each 5 μM) or DMSO for 6 h prior to stimulation with LPS (1 μg, 30 m). Representative western blot series from one independent donor of three quantified, with adjusted density representing fold change of phosphorylated target staining, normalized to GAPDH staining, relative to the stimulated control (LPS). **b** Monocytes from four donors were pretreated as in **a**, stimulated with LPS (1 μg, 24 h), and supernatant cytokine content was quantified on the MSD platform. **c**–**e** PBMCs from three donors were pretreated with AZD2014 for 6 h and stimulated with LPS (1 ng, 18 h) prior to staining. Representative flow plot (**c**) and aggregates (**d** and **e**) represent gating on leukocyte/singlet/live/CD14^+^. Boolean gating (**e**) facilitated identification of subpopulations producing different combinations of cytokines. **e** Illustrates that some monocytes produced no cytokines at all (turquoise), whether unstimulated, only stimulated with LPS, or stimulated with LPS after mTORi pretreatment. In contrast, the color coding described in the rightmost panel in **e** indicates that others produced one cytokine or different combinations of two or three cytokines, with “polyfunctionality” being defined as production of 2 or 3 cytokines in different combinations. Enahnced polyfunctionality was seen among the mTORi pretreated, LPS-stimulated monocytes. Statistical analyses: for the blot quantification (**a**), significance was determined using an ANOVA with Dunnett’s multiple comparisons, and asterisks (*) indicate a comparison to the stimulated control. For **b**, significance was determined via one-way ANOVA Tukey’s multiple comparisons, except in cases where Friedman tests and Dunn’s multiple comparisons were appropriate. For **d**, asterisks (*) indicate comparisons between US and LPS/mTORi (AZD2014) and hashes (#) indicate comparisons between LPS and LPS/mTORi (AZD2014), and significance was computed via two-way ANOVA and Tukey’s multiple comparisons. */# *p* < 0.05, **/## *p* < 0.01, ***/### *p* < 0.001. Error bars represent mean ± SD. For **e**, cytokine profiles were compared using Chi-square tests.

To document the efficacy of suppression of mTOR activity in LPS-stimulated primary human monocytes isolated from peripheral blood mononuclear cells (PBMC), we probed for phosphorylated species of downstream mTOR targets ribosomal protein S6 and eukaryotic translation initiation factor 4E-binding protein 1 (4E-BP1) with and without pretreatment with two structurally distinct mTORi and the mTORC1 allosteric inhibitor, rapamycin (Fig. 1a). Both mTORi surpassed rapamycin in suppression of phosphorylation of these targets, which are critical to initiate translation downstream of mTOR activation. Despite the inhibition of these activities of mTOR that enable protein synthesis, and consistent with an earlier report^25^, pretreatment of monocytes with mTOR inhibitors did not decrease production of pro-inflammatory cytokines, including IL-1β and IL-6, following LPS exposure (Fig. 1b). Potentiation of IL-1β release by LPS after pretreatment with one mTORi studied here (AZD2014) exceeded that observed with LPS alone (Fig. 1b) and that mTORi was selected for further study. The same mTORi boosted IL-6 secretion by monocytes following LPS stimulation; LPS stimulation alone yielded variable increases in IL-6 production that did not reach significance (Fig. 1b). Pretreatment with other mTOR inhibitors trended similarly to AZD2014 (Fig. 1b). Increased IL-10 production, relative to unstimulated monocytes, was seen with LPS alone (Fig. 1b), but this anti-inflammatory cytokine was not increased by LPS following mTORi pretreatment. Pretreatment with either rapamycin or mTORi similarly suppressed LPS-mediated IL-10 increases, suggesting that this decrease in an anti-inflammatory cytokine may occur via mTORC1 (Fig. 1b). Neither rapamycin nor an mTORi suppressed TNF-α elicited by LPS stimulation in these experiments (Fig. 1b).

To confirm results from isolated monocytes with an independent method, we next performed intracellular cytokine staining of LPS-stimulated PBMC and gated on monocytes (Fig. 1c). Results confirmed that mTORi pretreatment enhanced IL-1β and IL-6 production by CD14^+^ cells exposed to LPS, relative to LPS exposure alone (Fig. 1c, d). Boolean gating analysis of cytokine production among these monocytes also revealed remarkably enhanced functionality (defined here as the number of different cytokines produced) in the context of mTOR inhibition. Monocytes among LPS-stimulated PBMC produced all three pro-inflammatory cytokines (IL-1β, IL-6, and TNF-α), representing 31.4% of the total monocyte population that was pretreated with an mTORi versus 0.8% among the control cells exposed only to LPS (Fig. 1e). Together, these data show that mTORi pretreatment of monocytes stimulated with LPS ex vivo result in robust increases in polyfunctionality and, particularly, production of IL-1β and IL-6 (Fig. 1c–e).

### Inhibition of mTOR activity potentiated LPS-induced surface expression of TF on monocytes

**Fig. 2:**
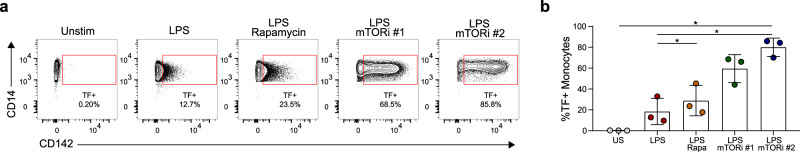
mTOR inhibition potentiates surface expression of tissue factor on primary uninfected human monocytes following TLR4 stimulation. **a**, **b** Freshly isolated human monocytes from three independent donors were pretreated as indicated with rapamycin (100 nM) or one of two structurally distinct mTORi (AZD2014, mTORi #1; INK128, mTORi #2; each 5 μM) or DMSO for 6 h and stimulated with LPS (1 μg, 12 h) prior to staining Representative flow plot (**a**) and aggregate (**b**) represent gating on leukocyte/singlet/live/CD14^+^. For **b**, significance was determined via one-way ANOVA and Tukey’s multiple comparisons. *p < 0.05. Error bars represent mean ± SD.

Given the enhanced functionality of mTORi-pretreated monocytes demonstrated above (Fig. 1e), we tested whether mTORi pretreatment also modulated LPS-stimulated monocyte surface TF expression, a known driver of HIV coagulopathy even during viremia-suppressing ART and a marker associated with inflammatory, polyfunctional monocytes in HIV and SIV infection^11^. mTORi pretreatment led to significant increases in the proportion of TF-positive monocytes following LPS stimulation, as compared to DMSO-pretreated controls (Fig. 2a, b).

### Inhibition of mTOR enhanced LPS-induced pro-inflammatory and pro-coagulant transcriptional responses

**Fig. 3:**
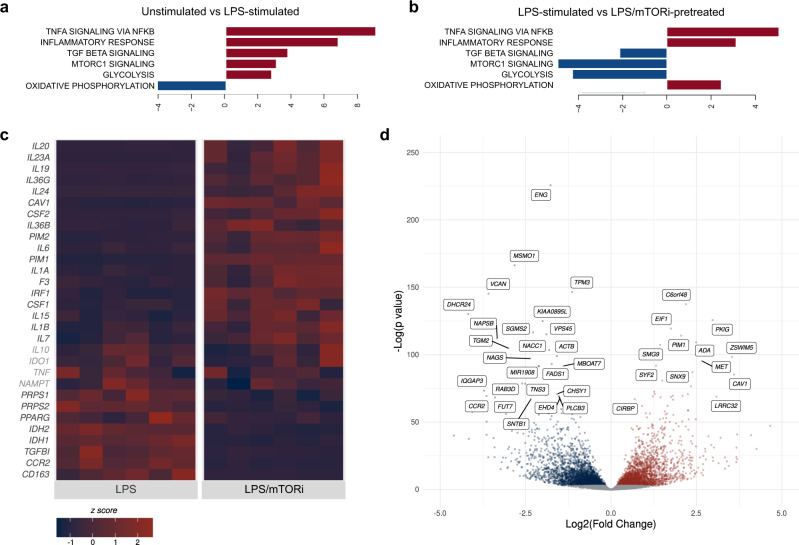
mTOR inhibition of LPS-stimulated monocytes promotes an inflammatory transcriptional program. **a**–**d** Monocytes from six independent donors were pretreated with an mTORi (AZD2014 at 5 μM, 6 h) or DMSO and stimulated with LPS (1 ng, 6 h) for bulk RNA-seq analysis. Select HALLMARK gene sets identified as differentially regulated through GSEA, presenting expression data comparing LPS-stimulated monocytes to unstimulated monocytes (**a**) and mTORi-treated, stimulated monocytes to LPS-stimulated monocytes without mTORi pretreatment (**b**)^81^. For complete HALLMARK GSEA, see also Supplementary Fig. 4. **c**, **d** Differential expression of genes of interest. **c** Expression normalized by z score; genes that did not reach significance are noted in light gray. **d** Significantly upregulated (red) or downregulated (blue) genes determined using an FDR adjusted *p* value cut-off of 0.05. Top 50 genes by FDR adjusted *p* value labeled.

RNA sequencing was employed to independently evaluate the differential expression associated with LPS stimulation after mTORi pretreatment. Monocytes from six independent donors were each treated under three conditions. Monocytes were left unstimulated or stimulated with LPS ex vivo following either DMSO or mTORi pretreatment. Comparison of LPS-stimulated monocytes to unstimulated, paired samples using gene set enrichment analysis (GSEA) showed that mTORC1 signaling, and the glycolytic metabolic program it supports, were enhanced after LPS stimulation (Fig. 3a). Expression of genes associated with oxidative phosphorylation (OXPHOS) decreased after LPS exposure, relative to unstimulated cells (Fig. 3a). Changes in enrichment among the mTORC1, glycolysis, and OXPHOS programs observed with LPS stimulation were reversed by mTORi pretreatment (Fig. 3a, b). LPS stimulation yielded the most significant enrichment among genes related to ‘TNF signaling via NF-κB’ and the ‘inflammatory response’ (Fig. 3a, Supplementary Fig. 1a). Notably, pretreatment with an mTORi, relative to DMSO, resulted in additional enrichment of these two gene sets after LPS stimulation (Fig. 3b, Supplementary Fig. 1b).

Differential analysis also revealed significantly increased abundance of transcripts mapping to genes encoding IL-1β, IL-6, and TF (*F3*) following mTORi pretreatment, relative to LPS stimulation alone (Fig. 3c). Transcripts of additional pro-inflammatory mediators augmented by mTOR inhibition included *IL1A*, *IL15*, *IL23A*, *CSF2*, and *IRF1*. In addition, mTORi pretreatment, relative to DMSO pretreatment, downregulated monocyte expression of *CCR2* and *CD163* (Fig. 3c, d). Transcripts associated with ‘cholesterol homeostasis’ were also suppressed in mTORi-treated, LPS-stimulated monocytes, relative to DMSO-pretreated, LPS-stimulated monocytes (Supplementary Fig. 1b), consistent with prior reports linking macrophage cholesterol depletion with pro-inflammatory cytokine production^26,27^. In contrast, mTORi was not found to transcriptionally modulate *TNF* expression among LPS-stimulated monocytes (Fig. 3c). These results align with observations at the protein level in presented in Fig. 1, and further suggest involvement of a transcriptional mechanism that couples with well-established translational pathways governed by mTOR.

### mTORi-mediated enhancement of surface TF expression among LPS-stimulated monocytes was NF-κB dependent

Promoters for *IL1B*, *IL6*, and *F3*, the gene encoding TF, bear κB binding sites. Thus, we tested mTORi effects on monocyte NF-κB activity. We found that mTORi did not decrease LPS-induced NF-κB activity in monocytes (Supplementary Fig. 2a). We next tested if NF-κB activity was required for enhanced *F3* expression. We observed a significant diminution in the frequency of TF-expressing monocytes when an inhibitor of IκB kinase (ACHP) was added to pretreatment with an mTORi prior to LPS stimulation, relative to only mTORi pretreatment (Supplementary Fig. 2b, c). We noted also that surface expression of CD14 on monocytes is exquisitely sensitive to ACHP treatment (Supplementary Fig. 2d, e). Consequently, gating of monocytes in analysis of this experiment relied on CD64 expression. CD14 engagement is not strictly required for LPS to signal via TLR4 through downstream MyD88-dependent processes, although it is required for TRIF-IRF axis signaling after TLR4 engagement^28^. Consistent with reports that PD-L1 and CD80 are also inducible through NF-κB^29,30^, ACHP also diminished their expression (Supplementary Fig. 2f, g). Additionally, an independent experimental approach using conventional ChIP analysis identified increased occupancy of the NF-κB p65 subunit at κB sites in the promoter regions of *F3* and *IL6* with mTORi pretreatment, beyond that observed with LPS stimulation alone (Supplementary Fig. 2h). Amplification from DNase I-insensitive regions far upstream of either locus’ transcriptional start sites (TSS) did not show a similar degree of enrichment (Supplementary Fig. 2h).

### Metabolomic analyses indicated that mTORi depletes NAD^+^ by limiting salvage pathway synthesis

**Fig. 4:**
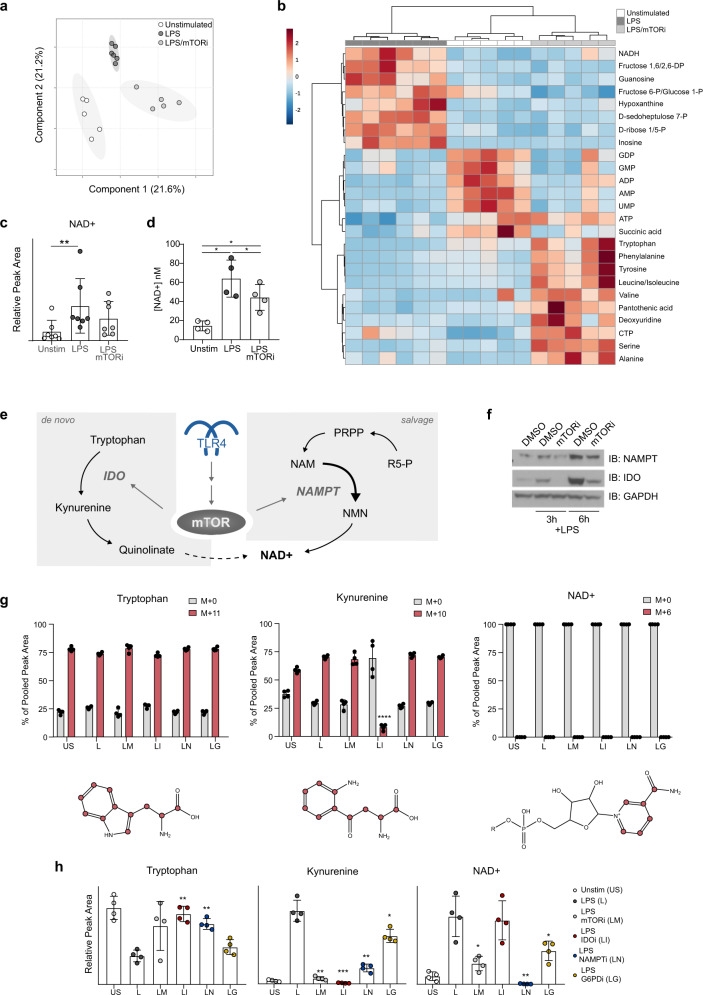
mTOR inhibition restricts NAD^+^ synthesis in LPS-stimulated primary human monocytes. **a**–**c** Donor monocytes (*n* = 7) were pretreated with an mTORi (AZD2014 at 5 μM, 6 h) or vehicle (DMSO) and stimulated with LPS (1 ng, 18 h) prior to metabolome extraction and analysis via LC-MS. **a** Partial least squares discriminant analysis (PLS-DA) and **b** hierarchical clustering analysis of “top 25” metabolites identified via one-way ANOVA performed using MetaboAnalyst^83^. **c** Shows relative peak area of the individual metabolite NAD^+^. Significance was assessed with a Friedman test and Dunn’s multiple comparisons. **d** Monocytes from four independent donors were pretreated and stimulated ex vivo as in **a** and intracellular NAD^+^ content was quantified differently using an enzymatic assay. Significance was evaluated by one-way ANOVA and Tukey’s multiple comparisons. **e** Schematic outlining pathways supporting NAD^+^ biosynthesis. **f** Donor monocytes (*n* = 3 unique donors) were pretreated as in **a** and stimulated with LPS (1 ng) for the specified duration (3 or 6 h) prior to lysis and western analysis. Representative blot series from one donor of three shown. G-H Donor monocytes (*n* = 4 unique donors) were pretreated as indicated with inhibitors of either mTOR (AZD2014) (LM, light gray), IDO1 (epacadostat) (LI, red), NAMPT (FK866) (LN, blue), or G6PD (G6PDi-1) (LG, yellow), cultured in media supplemented with ^13^C-tryptophan for 6 h and then stimulated with LPS (1 ng, 18 h). Note that key to abbreviations of conditions is listed in panel **h** for conditions used in **g** and **h**. **g** The metabolome was extracted and targeted analysis was obtained via LC-MS. The abundance of the unlabeled (M + 0, gray columns) and labeled (maroon columns) species of interest are shown as a percentage of the total abundance of each metabolite. Representations of labeled species with each ^13^C marked in maroon are under graphs in **g**. **h** Shows relative peak area of three metabolites of interest: tryptophan, kynurenine, and NAD^+^. For **g** and **h**, significance was determined by two-tailed *t*-tests, comparing LPS-induced response after each inhibitor pretreatment to the DMSO pretreatment, LPS-stimulated condition. For **g**, relative abundances of only the labeled species of kynurenine (in maroon) were compared, evaluating LPS-induced response after each inhibitor pretreatment compared to the DMSO pretreatment, LPS-stimulated condition. Statistical evaluation was not performed for tryptophan or NAD^+^ in **g**. *p < 0.05, **p < 0.01, ***p < 0.001, ****p < 0.0001. Error bars represent mean ± SD in any panel showing them.

We next studied the effects of mTORi pretreatment and LPS stimulation using steady-state metabolomics. We compared LC-MS-based hydrophilic metabolite profiling of lysates of monocytes isolated from seven independent donors that were each either usntimulated by LPS, DMSO-pretreated/LPS-stimulated, or mTORi-pretreated/LPS-stimulated. Partial least squares discriminant analysis (PLS-DA) of the clean data set found significant class differences, discriminating among the three treatment conditions (Fig. 4a). Distinct differences among these three conditions in major metabolities included an increased abundance of glycolytic intermediates observed among DMSO-pretreated, LPS-stimulated monocytes that was not observed among mTORi-pretreated, LPS-stimulated monocytes (Fig. 4b). This provides internal validation that mTORi pretreatment prevents an LPS-induced shift towards increased glucose utilization, but does not readily explain how mTORi can increase NF-κB-dependent transcription with LPS stimulation. However, we noted that intracellular pools of NAD^+^, a critical cofactor for activity of sirtuins that can down-modulate transcription via deacetylation of both histone and non-histone targets^31^, expanded following LPS stimulation and that mTORi may have restricted this expansion (Fig. 4c). Quantification of the absolute NAD^+^ concentration of monocyte lysates from four different donors (not used in experiments documented in Fig. 4a–c) with an independent method using an enzymatic assay added support (Fig. 4d). DMSO-pretreated, LPS-stimulated monocytes had a higher intracellular concentration of NAD^+^ than did mTORi-pretreated, LPS-stimulated monocytes in that assay (Fig. 4d).

Both de novo synthesis from tryptophan and salvage from either pentose phosphate pathway (PPP)-synthesized ribulose 5-phosphate (R5-P) or other sources of nicotinamide (NAM) can contribute to NAD^+^ pools (Fig. 4e). Immunoblots showed mTOR-dependent upregulation of the rate limiting enzymes in each of these two pathways, indolamine 2,3-dioxygenase (IDO) and nicotinamide phosphoribosyltransferase (NAMPT), in monocytes stimulated with LPS after DMSO (Fig. 4e, f). Although mTORi pretreatment decreased cellular abundance of both of these proteins after LPS for 3 or 6 h (Fig. 4f), transcription of *IDO1* and *NAMPT* were not differentially regulated in comparison of mTORi-pretreated, LPS-stimulated monocytes to DMSO-pretreated, LPS-stimulated monocytes (Fig. 3c). This suggested translational or post-translational mechanism(s) downstream of mTOR underlying the observed decreases in IDO1 and NAMPT (Fig. 4f), and prompted study of contribution of each pathway.

To determine whether the de novo NAD^+^ synthesis pathway contributed to the observed increase in NAD^+^ after LPS stimulation for 18 h, we performed targeted analysis of tryptophan metabolism using ^13^C-labeled tryptophan (Fig. 4g). More than 75% of tryptophan was labeled in monocytes that were either unstimulated; DMSO-pretreated, LPS-stimulated; mTORi-pretreated, LPS-stimulated; IDO inhibitor-pretreated, LPS-stimulated; NAMPT inhibitor-pretreated, LPS-stimulated; or glucose-6-phosphate dehydrogenase (G6PD) inhibitor-pretreated, LPS-stimulated (Fig. 4g). We observed accumulation of tryptophan-derived ^13^C in kynurenine among LPS-stimulated monocytes, more so than among unstimulated monocytes (Fig. 4g). Accumulation of ^13^C in kynurenine was significantly impaired by IDO inhibitor pretreatment, documenting the functional activity of the IDO inhibitor; no other tested inhibitor diminished the increase in ^13^C-labled kynurenine after LPS stimulation (Fig. 4g). In contrast, no accumulation of ^13^C was seen in cellular NAD^+^ under any tested condition, demonstrating that tryptophan-derived carbons are not utilized for NAD^+^ synthesis in this system (Fig. 4g). This indicates the predominance of the salvage pathway in increasing intracellular NAD^+^ after LPS stimulation of monocytes.

We next independently interrogated the contribution of each pathway to the LPS-induced increase in NAD^+^ by pretreatment with inhibitors specific for either IDO in the de novo pathway, NAMPT in the salvage pathway, or G6PD catalyzing the first step in the PPP providing precursors for the salvage pathway. Pretreatment with an inhibitor of IDO did not restrict the increase in NAD^+^ pools following LPS-stimulation (Fig. 4h), consistent with the ^13^C-labeled tryptophan experiment. In contrast, we found severe restriction of LPS-stimulated increased monocyte NAD^+^ levels with pretreatment with an inhibitor of the rate-limiting enzyme in the salvage pathway, NAMPT (Fig. 4h). There was a lesser, statistically significant blunting of the NAD^+^ increase with a G6PD inhibitor (Fig. 4h). Expression of genes encoding the phosphoribosyl pyrophosphate synthetases (*PRPS1* and *PRPS2*) was also suppressed by mTORi pretreatment, relative to DMSO-pretreated, LPS-stimulated controls (Fig. 3c). This is consistent with mTORi-restricted flow through the PPP in monocytes, as we previously reported in CD4 T cells^18^, that can also contribute to diminished NAD^+^ availability via the salvage pathway with mTORi pretreatment before LPS. mTORi pretreatment was also repeated and showed reproducible inhibition of LPS-induced increased NAD^+^ (Fig. 4h). Both the ^13^C-labeled tryptophan experiment (Fig. 4g) and the inhibitor experiment (Fig. 4h) indicate that mTORi pretreatment decreases NAD^+^ via effects on the salvage pathway.

LPS stimulation, which was shown above to enhance intracellular NAD^+^ pools, was associated with increased monocyte mitochondrial membrane potential (Supplementary Fig. 3a). Inhibition of either mTOR or NAMPT prior to LPS each prevented this increased monocyte mitochondrial membrane potential, whereas pretreatment with a selective IDO inhibitor did not (Supplementary Fig. 3a). None of these three inhibitors affected mitochondrial mass over the 24 h experiment (Supplementary Fig. 3b).

### Inhibition of mTOR potentiated surface TF expression equivalently among LPS-stimulated monocytes from chronically SIV-infected and uninfected rhesus macaques

**Fig. 5:**
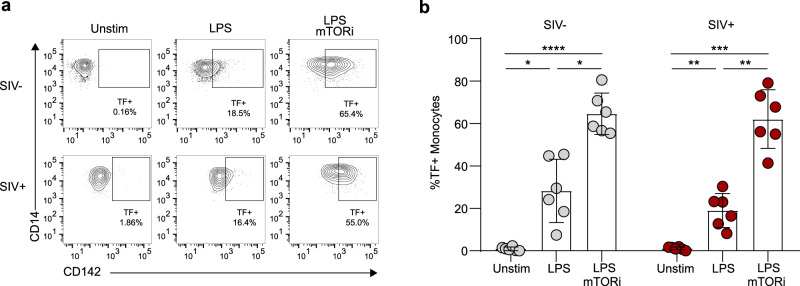
mTOR inhibition potentiates surface expression of tissue factor on monocytes from uninfected and SIV-infected macaques in an equivalent manner. **a**, **b** Cryopreserved PBMCs from six uninfected and six chronically SIVmac239-infected rhesus macaques were pretreated with an mTORi (AZD2014 at 5 μM, 6 h) or DMSO and stimulated with LPS (1 ng, 12 h) prior to staining for flow cytometry. For **b**, gray dots represent uninfected (SIV−) animals and red dots represent infected (SIV+). Representative flow plots (**a**) and aggregate (**b**) represent gating on leukocyte/singlet/live/CD11b^+^/CD14^+^. For additional details on gating strategy see Supplementary Fig. 6. *p < 0.05, **p < 0.01, ***p < 0.001, ****p < 0.0001, *p* values calculated using a two-way ANOVA and Sidak’s multiple comparisons. Error bars represent mean ± SD.

Pathogenic simian immunodeficiency virus (SIV) infection of macaques is an established model of HIV infection. SIV-infected and -uninfected rhesus macaque (*Macaca mulatta)* PBMCs were studied ex vivo here. Blood CD4/CD8 lymphocyte counts, viral load, and plasma cytokines were assessed at time of PBMC collection from the macaques (Supplementary Fig. 4a–d). These SIVmac239-infected animals showed varying degrees of disease progression, though all were chronically infected and had not progressed to AIDS. Pretreatment with mTORi before LPS stimulation of PBMCs ex vivo increased the proportion of TF-expressing monocytes from both uninfected and chronically SIVmac239-infected macaques relative to DMSO-pretreated, LPS-stimulated monocytes (Fig. 5a, b), paralleling our findings in human primary monocytes. There was no difference in the proportion of surface TF-expressing monocytes among PBMCs from SIV-infected versus uninfected animals under any condition tested (Fig. 5a, b).

## Discussion

Chronic systemic inflammation likely accelerates a range of cardiovascular pathologies in PLWH on ART, including diatolic dysfunction, scarring following myocardial infarct, and stroke^4^. Inflammation is sustained during ART by multiple mechanisms, including pro-inflammatory and pro-coagulant mediators released by myeloid cells in response to translocated microbial products^32^. Given that LPS-activated TLR4 signaling in monocytes activates mTOR to coordinate metabolic reprogramming, inhibition of mTOR has been hypothesized as a potential intervention. There are also reports that metabolically-activated monocytes from ART-suppressed PLWH more readily migrate across endothelia to generate foam cells in an ex vivo model of atherogenesis^22^. The success of sirolimus-eluting stents for treating coronary artery disease (CAD)^33^, the documented anti-HIV effects of mTORi^15–18,34,35^, and their potential to reduce HIV reservoirs^36–39^ have added to interest in testing mTOR inhibitors as an adjunct to ART that may decrease monocyte-derived inflammatory mediators to ameliorate CVD risk in PLWH^24^.

However, some reports show mTOR inhibition may increase inflammation. A preliminary report of a trial of adjunctive sirolimus in ART-suppressed PLWH found that plasma biomarkers of systemic inflammation were increased, despite benefits in decreasing immune exhaustion and provirus load^40^. Inhibition of mTOR also enhanced procoagulant TF activity on murine peritoneal macrophages^41^ and increased NF-κB transcriptional activity in endothelial cells^42^. Also relevant is a study of human monocytes in which Weichhart et al. demonstrated that rapamycin potentiated their production of IL-12 in response to LPS ex vivo, and also increased NF-κB transcriptional activity^25^. However, that report did not note monocyte culture conditions. We have found that commonly used tissue culture plates cause contact-induced monocyte activation, as has been previously shown^43^, raising the possibility that there could have been an additional pathway to TLR4 signaling by which mTOR was activated in that study^25^. We documented that PTFE-coated plate inserts (Millipore Sigma) prevented contact-induced activation of the monocytes isolated by bead-based negative-selection and exclusively used them in all experiments reported here to ensure activation only by LPS.

The results here show that catalytic mTOR inhibition of primary human monocytes increased LPS-stimulated pro-inflammatory cytokine and TF transcriptional responses in primary human monocytes. Catalytic mTOR inhibitors led to a more accentuated pro-inflammatory and pro-coagulant response to LPS than did a rapalog (Figs. 1b and 2b), consistent with additional effects of mTORi on reversing anti-inflammatory mTORC2-Akt signals^44,45^. The use of these catalytic inhibitors targeting both mTOR complexes enhances the novelty of this work and extends beyond the one earlier report^25^. In depth analyses here focused on one mTORi (AZD2014), but similar responses seen with two mTORi and rapamycin support that this is a class-wide effect and not specific to a single inhibitor.

This work is limited by its consideration of the total monocyte population only, as opposed to disambiguation of responses among monocyte subtypes. This affords the benefit of approximating the in vivo monocyte population, which may offset the lost opportunity to pinpoint relevant subtypes. The implictions of the results here for mTORi to be used as a clinical treatment hold irrespective of which monocyte subtype may be primarily responsible for its effect to increase monocyte-derived pro-inflammatory and pro-coagulant responses after LPS.

RNA sequencing analyses conclusively supported our flow cytometry results and indicated a transcriptional mechanism underlying enhanced production of IL-1β, IL-6, and TF here. Combining treatment with an inhibitor of IκB kinase (ACHP) and an mTORi before LPS stimulation significantly diminished the potentiation of surface TF seen with pretreatment with only an mTORi. This demonstration of an NF-κB-dependent effect on transcription was supported by the observation of increased p65 occupancy at the κB sites in the promoter regions of *F3* and *IL6* with mTORi pretreatment, and not at upstream, control sites. Another observation that is consistent with NF-κB-dependence of the pro-coagulant effect seen with mTOR inhibition: expression of the serine/threonine kinase *PIM1* and its semi-redundant isoform *PIM2* was increased among mTORi-pretreated monocytes (Fig. 3c, d); PIM1 has been reported to both stabilize p65^46^ and control reactivation of latent HIV proviruses^47^.

Increased TF expression seen here on mTORi-pretreated, LPS-stimulated, monocytes suggests a risk of heightening a hypercoagulable state that could precipitate vaso-occlusive events. An earlier report of in vivo treatment of SIV-infected pigtailed macaques with an anticoagulant supported the concept that inhibiting the extrinsic, TF-dependent coagulation pathway can significantly decrease D-dimer and immune activation in vivo, without adversely impacting monocyte responses to TLR stimulation^11^. Of note, the finding here that mTORi increased the proportion of TF-expressing rhesus macaque monocytes after LPS exposure ex vivo, as in humans, supports future study of potential interventions to limit this pro-coagulant effect in this animal model. The fact that this mTORi effect was similar here in cells from both SIV-infected and uninfected macaques differs from a report using a related species of pigtail macaques infected with SIVsabBH66, a strain different than the one used here, that was selected by serial passage for increased virulence^11,48^. These contrasting results from different species and viruses suggest that studying both SIV-infected and uninfected animals may be optimal for future in vivo efforts to assess interventions to limit pro-coagulant responses to mTORi.

In contrast to the enhanced expression of *F3* and pro-inflammatory cytokines, we observed suppression of *CCR2* and *CD163* expression with mTORi pretreatment (Fig. 3c), raising a hypothesis that mTORi may limit monocyte transmigration to the intima, even if enhanced TF expression is a separate factor increasing risk of vaso-occlusion on LPS exposure.

Metabolomic analyses here led to a hypothesized mechanism for mTORi-mediated NF-κB-dependent transcriptional upregulation of these pro-inflammatory and pro-coagulant genes that supports a role for mTOR in resolving this acute inflammatory response. LC-MS-based hydrophilic metabolite profiling suggested, and an independent method confirmed, that intracellular pools of NAD^+^ expanded following LPS stimulation and that mTORi restricted this expansion. Results here also show that mTORi blocks the increase in mitochondrial membrane potential after LPS seen here (Supplementary Fig. 3a) and earlier^49^. This is consistent with the pivotal role of NAD^+^/NADH in supporting mitochondrial function and extends earlier work by implicating mTOR in the mechanism underlying this previously reported effect of LPS on mitochondria^49^. NAD^+^ is also a critical cofactor essential for activity of the sirtuin family of deacetylases, which down-modulate NF-κB-dependent transcription by deacetylation of both histone and/or non-histone targets^31^. LPS-enhanced intracellular NAD^+^ may support sirtuin-mediated deacetylation at p65 K310; persistent acetylation at p65 K310 enhances inflammatory gene expression induced by LPS^50–54^. As noted before, we found increased occupancy of the NF-κB p65 subunit at κB sites in the promoter regions of *F3* and *IL6* with mTORi pretreatment, beyond that observed with LPS stimulation alone (Supplementary Fig. 2h). In addition, transcriptional activity at κB sites may be attenuated by p65-interacting sirtuin deacetylation of histone H3 lysine 9 (H3K9) at NF-kB target gene promoters^55^. Since a link to mTOR involvement in such immune responses has not previously been made, we explored how mTORi pretreatment impacted a global assessment of the transcription-activating mark, acetylated H3K9, after LPS stimulation. An accumulation of global acH3K9 was observed with mTORi treatment prior to LPS stimulation that could not be attributed to decreased methylation at H3K9 (Supplementary Fig. 6a–d). However, this experiment is not conclusive and more definitive address of potential sirtuin-dependent mechanisms is required; specifically, unbiased, genome-wide and confirmatory gene-targeted analyses are appropriate to rigorously evaluate mechanisms by which mTOR inhibition impacts NAD^+^-dependent epigenetic programming of monocytes. This includes evaluation of the alternative hypothesis that consumption of NAD^+^ may increase in mTORi pretreated, LPS-stimulated monocytes, as well as the hypothesis supported by results here of mTORi-mediated decreases in NAD^+^ synthesis^1^.

In addition, future work could explore whether other NAD^+^-related mechanisms may contribute to the transcriptional profile of the mTORi-pretreated, LPS-stimulated monocytes. For example, increased abundance of inflammatory transcripts observed here may reflect increased mRNA stability, as well as higher rates of transcription^56,57^. Modulation of the activity of RNA-binding proteins (RBP) through ADP-riboslyation, a NAD^+^-intensive process, may contribute to the enhanced inflammatory program observed among mTORi-treated monocytes. As demonstrated by by Ke et al., modification of the RBP HuR by poly-(ADP) ribosylase (PARP1) regulates the translation of inflammatory cytokines and chemokines in LPS-stimulated macrophages^57^. Of note, the *F3* locus encodes an RBP-target adenylate-uridylate-rich element (ARE) at the 3’ UTR, also suggesting study of a role for NAD^+^-dependent PARylation in the coagulopathic phenotype exhibited by mTORi-pretreated, LPS-stimulated monocytes. Such a contribution of dysregulated PARP activity to enhancement of both NF-kB activity and oxidative damage was previously shown in cardiomyocytes and murine macrophages^58–60^.

Recent evidence indicates that extracellular NAMPT and extracellular NAPRT can each be endogenous ligands for TLR4, capable of activating NF-κB in a manner independent of their enzymatic activities^61,62^. Managó et al. also report an association between elevated levels of extracellular NAPRT (eNAPRT) and reduced survival among septic patients and suggest the use of eNAPRT as a clinical marker^62^. This prompts consideration of these noncanonical TLR4 ligands as potential markers of immune activation in HIV infection and, beyond this, exploration of a potential mechanistic role in accelerated CVD and other age-related conditions in PLWH. However, the finding here that intracellular NAMPT was decreased, not increased, by mTORi suggests that these noncanonical TLR4 ligands are not likely to explain the enhanced pro-inflammatory and pro-coagulant effects seen here. However, an unanticipated, paradoxical effect of mTORi to increase extracellular release of NAMPT or NAPRT cannot be excluded.

We next sought to determine whether de novo synthesis contributed to the increase in NAD^+^ by tracking flux of ^13^C-labeled tryptophan after LPS stimulation. Results indicated that tryptophan-derived carbons are not utilized for de novo synthesis of the bulk of this LPS-induced expansion of the NAD^+^ pool under the conditions studied here (Fig. 4g**)**. One limitation of this analysis is that a contribution of a small proportion of NAD^+^ from de novo synthesis cannot be excluded since 25% of the carbons in the ^13^C-labeled-tryptophan remained unlabeled (Fig. 4g). However, other independent approaches confirmed the predominance of the salvage pathway in increasing intracellular NAD^+^ very soon after LPS stimulation of monocytes, and in restriction of this increase by mTORi-pretreatment. Inhibition of IDO did not impede the increase in monocyte NAD^+^ (Fig. 4h) or the increase in mitochiondrial membrane potential (Supplementary Fig. 3a), each observed following LPS stimulation. Inhibition of the rate-limiting enzyme, NAMPT, in the salvage pathway for NAD^+^ synthesis profoundly restricted the rapid expansion of the NAD^+^ pool following LPS stimulation of monocytes (Fig. 4h) and prevented LPS-induced increased mitochondrial membrane potential (Supplementary Fig. 3a). A lesser, statistically significant blunting of the LPS-sitmulated increase in monocyte NAD^+^ was seen following pretreatment with a G6PD inhibitor that diminished products of PPP available to enter the salvage pathway (Fig. 4h). Thus, the increase in the monocyte intracellular pool of NAD^+^ after LPS stimulation depends on mTOR impacting the salvage pathway rather than de novo synthesis (Fig. 4e, indicated by the bold arrow). Results here extend, and independently confirm, earlier work implicating rapid increases in NAD^+^ via the salvage pathway in resolution of LPS-induced, NF-κB-mediated monocyte inflammation via effects on sirtuins and NF-κB^49^.

The dominant role of the salvage pathway in LPS-induced and mTORi-restricted synthesis of NAD^+^ suggests future directions for more translational HIV-related research. Several dietary supplements can be studied for possible enhancement of salvage pathway synthesis in vivo, including nicotinic acid (vitamin B3), nicotinamide riboside (NR) and nicotinamide mononucleotide (NMN). In previous studies, supplementation with NR did not increase intracellular NAD^+^ in cell culture and murine models, and was efficiently hydrolyzed in cell cultures even with serum-free media^63,64^. Similarly, NR supplementation of cell cultures here did not decrease surface TF in mTORi-pretreated, LPS-stimulated monocytes (Supplementary Fig. 5c). We also found a small reversal of decreased surface TF with NMN in only one of several cultures with mTORi-pretreated, LPS-stimulated monocytes from different humans. This lack of reproducibility of NMN effect ex vivo may be related to variation in residual monocyte surface CD38-mediated degradation of NMN and/or activity of hydrolases degrading NMN in bovine serum present in the culture medium^64^. However, restoration of intracellular NAD^+^ is documented in animal models and humans using oral supplements of NR, NMN, or vitamin B3^65–67^. NR has been reported to increase intracellular NAD^+^ in PBMCs obtained from study participants taking this supplement orally, and it was well-tolerated in vivo^68^. Recent work reports that NR supplementation in vivo reduced proinflammatory cytokine expression in four uninfected humans with heart failure^69^. Thus, one next step for research on how to prevent chronic inflammation-associated HIV comorbidities may be evaluation of an orally bioavailable NAD^+^ precursor given with an mTORi for mitigation of the mTORi’s pro-inflammatory and pro-coagulant effects; this combination can be studied as an adjunct to ART in SIV-infected rhesus macaques. A potential benefit of use of mTORi as an adjunct to ART, if its effect to decrease salvage synthesis of NAD^+^ can be countered by dietary supplementation, is that it may also help address the problem of latent and reactivatable provirus requiring life-long ART^36–39^. Such an experimental approach that maintains NAD^+^ pools during mTORi may potentially also have relevance for studying other inflammatory conditions in addition to lentivirus infection. For example, serious COVID-19 is associated with both an impaired gut barrier and depletion of NAD^+^ pools^70–72^.

Results here show that mTOR activities rapidly constrain pro-inflammatory transcriptional responses to LPS. In addition to the potential future directions above, further characterization of the mechanisms downstream of mTOR may discover targets for new therapeutic approaches to chronic inflammatory disorders that would not require NAD^+^-restoring supplements. Short-term mTOR inhibition could also be further studied with the intent to enhance pro-inflammatory responses to LPS as an intervention to sustain anti-bacterial mechanisms needed for recovery in acute sepsis models^49,73^.

## Methods

### Animal care and ethics

Rhesus macaques (*Macaca mulatta*) were housed and cared in accordance with American Association for Accreditation of Laboratory Animal Care standards in AAALAC accredited facilities, and all animal procedures were performed according to protocols approved by the Institutional Animal Care and Use Committees of the National Institute of Allergy and Infectious Diseases under animal study protocol LVD26. Experimental animals were infected intravenously with 2000 TCID50 SIVmac239. For study animal details, see Supplementary Table 1.

### Cell culture

Peripheral blood mononuclear cells (PBMC) were isolated via density gradient centrifugation using Ficoll-Paque PLUS (GE Healthcare, Pittsburg, PA) from uninfected donors’ EDTA-coagulated leukopacks (Lifesource, Rosemont, IL and New York Blood Center, New York, NY). Monocytes were purified through negative selection via magnetic-assisted cell sorting (Pan-Monocyte Isolation Kit, Miltenyi Biotec, Bergisch Gladbach, Germany) and resuspended in complete RPMI plus 10% defined FBS (GE Healthcare) and penicillin/streptomycin. Using a within-donor design, monocytes were pretreated for six hours with an inhibitor of mTOR (either rapamycin (100 nM), AZD2014 (5 μM), or INK128 (5 μM)), an inhibitor of NAMPT (FK866 (100 nM)), an inhibitor of IDO1 (Epacadostat (1 μM)), or an inhibitor of G6PD^74^ (G6PDi-1 (100 μM)) (Cayman Chemical, Ann Arbor, MI) and stimulated with LPS isolated from *E. coli* O111:B4 (Millipore Sigma, Darmstadt, Germany) as indicated in figure legends. All culture was performed in PTFE-coated plate inserts (Millipore Sigma) to prevent contact-induced monocyte activation.

### High sensitivity cytokine quantification

Supernatants were recovered from monocyte pellets and stored at −80 °C prior to assay. Briefly, thawed supernatants/plasma samples were diluted 1:4 prior to analysis using the MSD® MULTI-SPOT Assay system (Meso Scale Diagnostics, Rockville, MD).

### Flow cytometry

Following recovery from culture, PBMCs or monocytes were washed once in ice cold PBS prior to staining with Live/Dead fixable red dead cell stain (Life Technologies, Carlsbad, CA) for 30 m at 4 °C. Cells were washed once in cold PBE (PBS with 0.5% BSA and 2 mM EDTA) and stained with antibodies for extracellular markers diluted in PBE for 30 m at 4 °C. For ICS, cells were treated with Cytofix/Cytoperm (BD Biosciences, San Jose, CA) for 20 m at 4 °C, washed, and stained with antibodies for intracellular markers at 1:100 for 30 m at 4 °C. Human Fc Block (BD Biosciences) was used in all staining buffers. For intracellular cytokine staining, *Brefeldin A* (Millipore Sigma) was added to the culture at 5 mg/mL 6 h prior to harvest. Single-stained controls were prepared using UltraComp eBeads (Invitrogen, Carlsbad, CA). Data was acquired on a BD LSR II cytometer (BD Biosciences). Compensation and gating were accomplished using FlowJo v10 (TreeStar, Ashland, OR). Gating strategies are included in the supplement (Supplementary Fig. 7). A complete list of conjugated clones may be found in Supplementary Table 2.

### Mitochondrial mass and membrane potential (ΔΨm) analysis by flow cytometry

Primary monocytes were cultured and treated as indicated. Thirty minutes before harvest, cells were incubated at 37 °C with 100 nM MitoTracker Green FM probe (Life Technologies) and 100 nM MitoView 633 (Biotium) for 30 m in dark to evaluate mitochondrial mass and ΔΨm, respectively. After harvest, cells were stained with SYTOX Blue dead stain (Life Technologies) as recommened by vendor to monitor cell viability. Data were acquired on a BD LSRFortessa (BD Biosciences). Analysis of data was accomplished as indicated above.

### Immunoblotting

Cell pellets were resuspended in RIPA buffer (ThermoFisher Scientific, Waltham, MA) with protease and phosphatase inhibitors at 1.0 × 10^6^ cells/μL and permitted to lyse on ice for 30 m. After normalization by protein concentration, lysates were denatured and separated via SDS-PAGE and transferred to nitrocellulose membrane using a semi-dry system. After blocking, blots were probed with primary antibodies at 4 °C overnight, diluted in SuperBlock (ThermoFisher Scientific). Membranes were then washed and probed with HRP-conjugated secondary antibodies (ThermoFisher Scientific) prior to addition of substrate, film exposure, and development. A list of primary antibodies used for blotting may be found in Supplementary Table 3.

### NAD^+^ quantification and hydrophilic metabolites profiling

Following recovery from culture, monocytes were placed on ice and counted. An aliquot (5 × 10^5^) of monocytes were assayed using the NADglo assay (Promega, Madison, WI) according to manufacturer’s instructions. Remaining monocytes were washed twice in ice cold normal saline (0.9% NaCl) prior to application of extraction solution (80% methanol v/v) cooled to −80 °C. Pellets were incubated in extraction solution at −80 °C and then subjected to three freeze/thaw cycles, vortexing 30 s after each thaw. Following extraction of the metabolome to solution, debris was pelleted at 20,000 × *g* for 15 m at 4 °C and supernatant transferred to a fresh tube for drying using SpeedVac. 50% acetonitrile was added to the tube for reconstitution following by overtaxing for 30 s. Sample solution was then centrifuged for 15 m at 20,000 × *g* and 4 °C and supernatant was collected for LC-MS analysis.

Metabolomics services yielding data presented in Fig. 4a–c were performed by the Metabolomics Core Facility at Robert H. Lurie Comprehensive Cancer Center of Northwestern University. Samples were analyzed by High-Performance Liquid Chromatography and High-Resolution Mass Spectrometry and Tandem Mass Spectrometry (HPLC-MS/MS). Specifically, system consisted of a Thermo Q-Exactive in line with an electrospray source and an Ultimate3000 (Thermo) series HPLC consisting of a binary pump, degasser, and auto-sampler outfitted with a Xbridge Amide column (Waters; dimensions of 4.6 mm × 100 mm and a 3.5 µm particle size). The mobile phase A contained 95% (vol/vol) water, 5% (vol/vol) acetonitrile, 20 mM ammonium hydroxide, 20 mM ammonium acetate, pH = 9.0; B was 100% Acetonitrile. The gradient was as following: 0 min, 15% A; 2.5 min, 30% A; 7 min, 43% A; 16 min, 62% A; 16.1–18 min, 75% A; 18–25 min, 15% A with a flow rate of 400 μL/min. The capillary of the ESI source was set to 275 °C, with sheath gas at 45 arbitrary units, auxiliary gas at 5 arbitrary units and the spray voltage at 4.0 kV. In positive/negative polarity switching mode, an m/z scan range from 70 to 850 was chosen and MS1 data was collected at a resolution of 70,000. The automatic gain control (AGC) target was set at 1 × 106 and the maximum injection time was 200 ms. The top 5 precursor ions were subsequently fragmented, in a data-dependent manner, using the higher energy collisional dissociation (HCD) cell set to 30% normalized collision energy in MS2 at a resolution power of 17,500. Data acquisition and analysis were carried out by Xcalibur 4.1 software and Tracefinder 4.1 software, respectively (both from Thermo Fisher Scientific).

Additional metabolomics experiments and flux analysis of ^13^C-labeled tryptophan presented in Fig. 4g, h and Supplementary Fig. 3a, b were performed as follows: Water-soluble metabolites were extracted from primary monocytes via −80 °C 80:20 methanol:water with a volume of 75 μL solvent per 1-million cells, vortexed, incubated on dry ice for 10 min, and centrifuged at 16,000 × *g* for 20 min, with the supernatant used for LC-MS analysis. Extracts were analyzed within 24 h by liquid chromatography coupled to a mass spectrometer (LC-MS). The LC–MS method involved hydrophilic interaction chromatography (HILIC) coupled to the Q Exactive PLUS mass spectrometer (Thermo Scientific). The LC separation was performed on a XBridge BEH Amide column (150 mm 3 2.1 mm, 2.5 mm particle size, Waters, Milford, MA). Solvent A is 95%: 5% H2O: acetonitrile with 20 mM ammonium bicarbonate, and solvent B is acetonitrile. The gradient was 0 min, 85% B; 2 min, 85% B; 3 min, 80% B; 5 min, 80% B; 6 min, 75% B; 7 min, 75% B; 8 min, 70% B; 9 min, 70% B; 10 min, 50% B; 12 min, 50% B; 13 min, 25% B; 16 min, 25% B; 18 min, 0% B; 23 min, 0% B; 24 min, 85% B; 30 min, 85% B. Other LC parameters are: flow rate 150 ml/min, column temperature 25 °C, injection volume 10 μL and autosampler temperature was 5 °C. The mass spectrometer was operated in both negative and positive ion mode for the detection of metabolites. Other MS parameters are: resolution of 140,000 at m/z 200, automatic gain control (AGC) target at 3e6, maximum injection time of 30 ms and scan range of m/z 75–1000. Data were analyzed via the MAVEN software, and isotope labeling was corrected for natural ^13^C abundance in the tracer experiments^75^.

### Isotope labeling

Human plasma-like medium (HPLM) with [U-^13^C]Trp (Cambridge Isotope Laboratories) was prepared as previously reported^76^ except without Trp and supplemented with isotopic Trp (25 μM). Isotope-labeled tryptophan HPLM was prepared and supplemented with 10% dialyzed FBS (Hyclone).Cells were cultured in isotopic Trp-containing medium for a total of 24 h prior to harvest and frozen at −80 °C prior to analysis.

### RNA sequencing

RNA was extracted from cell pellets using the RNeasy Mini Kit (Qiagen, Hilden, Germany). The stranded mRNA-seq was conducted in the Northwestern University NUSeq Core Facility. Briefly, total RNA examples were checked for quality using RINs generated from Agilent Bioanalyzer 2100. RNA quantity was determined with Qubit fluorometer. The Illumina TruSeq Stranded mRNA Library Preparation Kit was used to prepare sequencing libraries from 200 ng of high-quality RNA samples (RIN > 7). The Kit procedure was performed without modifications. This procedure includes mRNA enrichment and fragmentation, cDNA synthesis, 3’ end adenylation, Illumina adapter ligation, library PCR amplification and validation. lllumina HiSeq 4000 NGS Sequencer was used to sequence the libraries with the production of single-end, 50 bp reads.

The quality of DNA reads, in FASTQ format, was evaluated using FastQC. Adapters were trimmed and aligned to the human genome (hg38) using STAR^77^. Read counts for each gene were calculated using htseq-count in conjunction with a gene annotation file for hg38 obtained from Ensembl (http://useast.ensembl.org/index.html)^78^. Normalization and differential expression were calculated using DESeq2 that employs the Wald test^79^. The cutoff for determining significantly differentially expressed genes was an FDR-adjusted *p* value less than 0.05 using the Benjamini-Hochberg method. A Gene Set Enrichment Analysis (GSEA) was performed to identify significantly enriched gene sets among the gene expression results^80,81^.

### Chromatin immunoprecipitation

Primary human monocytes (2 × 10^7^) were recovered from culture and pelleted prior to crosslinking, performed first in DSG/PBS solution for 30 min at RT, and then in 1% formaldehyde for 10 min at RT, quenched with glycine solution. Dual-crosslinked cells were washed twice in ice cold PBS, pelleted, and stored at −80 °C.

Pellets were thawed on ice and membranes disrupted using a syringe in the presence of protease inhibitors. Chromatin was sheared using a Bioruptor (Diagenode, Denville, NJ). Shearing conditions were optimized for the cell type and number. Sheared chromatin was clarified by centrifugation and immunoprecipitated in duplicate using rabbit anti-p65 (Abcam, Cambridge, UK) or normal rabbit IgG (Cell Signaling Technologies). Chromatin was reserved for normalization (10% of input). Bound chromatin was pulled down using Protein A-agarose beads pre-blocked with salmon sperm DNA (Millipore Sigma) and then de-crosslinked using Chelex 100 and Proteinase K. DNA cleanup was accomplished using a MinElute Kit (Qiagen) according to manufacturer’s instructions. Quantitative PCR was performed with iTaq Universal SYBR Green Supermix (Bio-Rad Laboratories, Hercules, CA). Primers were designed using the UCSC Human Genome Browser^82^. Primer sequences may be found in Supplementary Table 4.

### Statistics and reproducibility

Analysis was performed using PRISM v8.0.1 (GraphPad Software, La Jolla, CA) and error bars represent mean ± SD unless otherwise noted. Replicates are biological, representing independent donors. Experiments were repeated at least three times. Sample size varied depending on methodology and is defined in figure legends. Normality of data series was assessed using the Shapiro–Wilk method. Unless otherwise stated, parametric analyses performed using a one-way ANOVA and Tukey’s multiple comparisons, using the Greenhouse–Geisser correcton. In lieu of a one-way ANOVA, given failure of a test of normality, Friedman tests and Dunn’s multiple comparisons were used. Statistical analysis of metabolomic data relied on the online R-based platform MetaboAnalyst^83^. Prior to statistical analysis, the metabolomic data set was cleaned by removing targets with less than 66% non-zero values, normalized to the median, and outliers were detected using a random forest based method. Differential gene expression was visualized using the R package *ggplot2*^84^.

### Reporting summary

Further information on research design is available in the Nature Research Reporting Summary linked to this article.

## Supplementary information

Supplementary Information

Description of Additional Supplementary Files

Supplementary Data 1

Supplementary Data 2

Reporting Summary

## Data Availability

All source data are available via the Northwestern University Digital Hub: https://digitalhub.northwestern.edu/collections/5f1b1739-512f-4015-98bc-22f37f42af7b?sort=label_si+asc The RNA sequencing data that support the findings of this study have been deposited in the NCBI GEO database (accession code: GSE187403). Metabolomic and metabolic tracing data can be found in Supplementary Data 1 and 2 files, respectively.
