# Supplementary material for: mTOR regulation of metabolism limits LPS-induced monocyte inflammatory and procoagulant responses
Source: Commun Biol. 2022 Aug 26;5:878. doi: 10.1038/s42003-022-03804-z (PMC9412771; doi:10.1038/s42003-022-03804-z)
Supplement: Supplementary file 1 — Supplementary Information [file 42003_2022_3804_MOESM1_ESM.pdf]

mTOR regulation of metabolism limits LPS-induced monocyte inflammatory and procoagulant responses

SUPPLEMENTARY FIGURES AND TABLES

Supplementary Figures

Supplementary Figure 1……………………………………………2
Supplementary Figure 2……………………………………………3
Supplementary Figure 3……………………………………………5
Supplementary Figure 4……………………………………………6
Supplementary Figure 5……………………………………………7
Supplementary Figure 6……………………………………………8
Supplementary Figure 7…………………………………………....9

Supplementary Tables

Supplementary Table 1……………………………………………10
Supplementary Table 2……………………………………………10
Supplementary Table 3……………………………………………11
Supplementary Table 4……………………………………………11

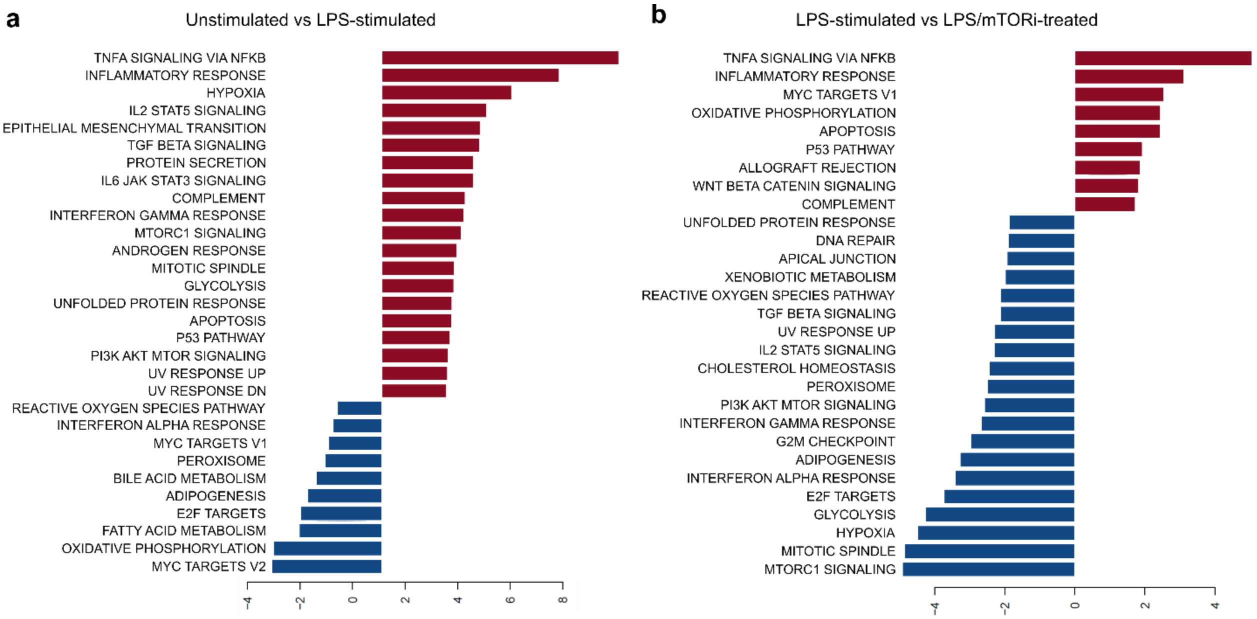

Supplementary Fig 1. Gene set enrichment analysis of bulk RNA-seq data. Enrichment among HALLMARK gene sets, comparing reads from unstimulated monocytes versus LPS-stimulated monocytes (A) and from comparison of reads from stimulated monocytes versus mTORi-treated, stimulated monocytes (B). Data represents paired samples from six independent donors.

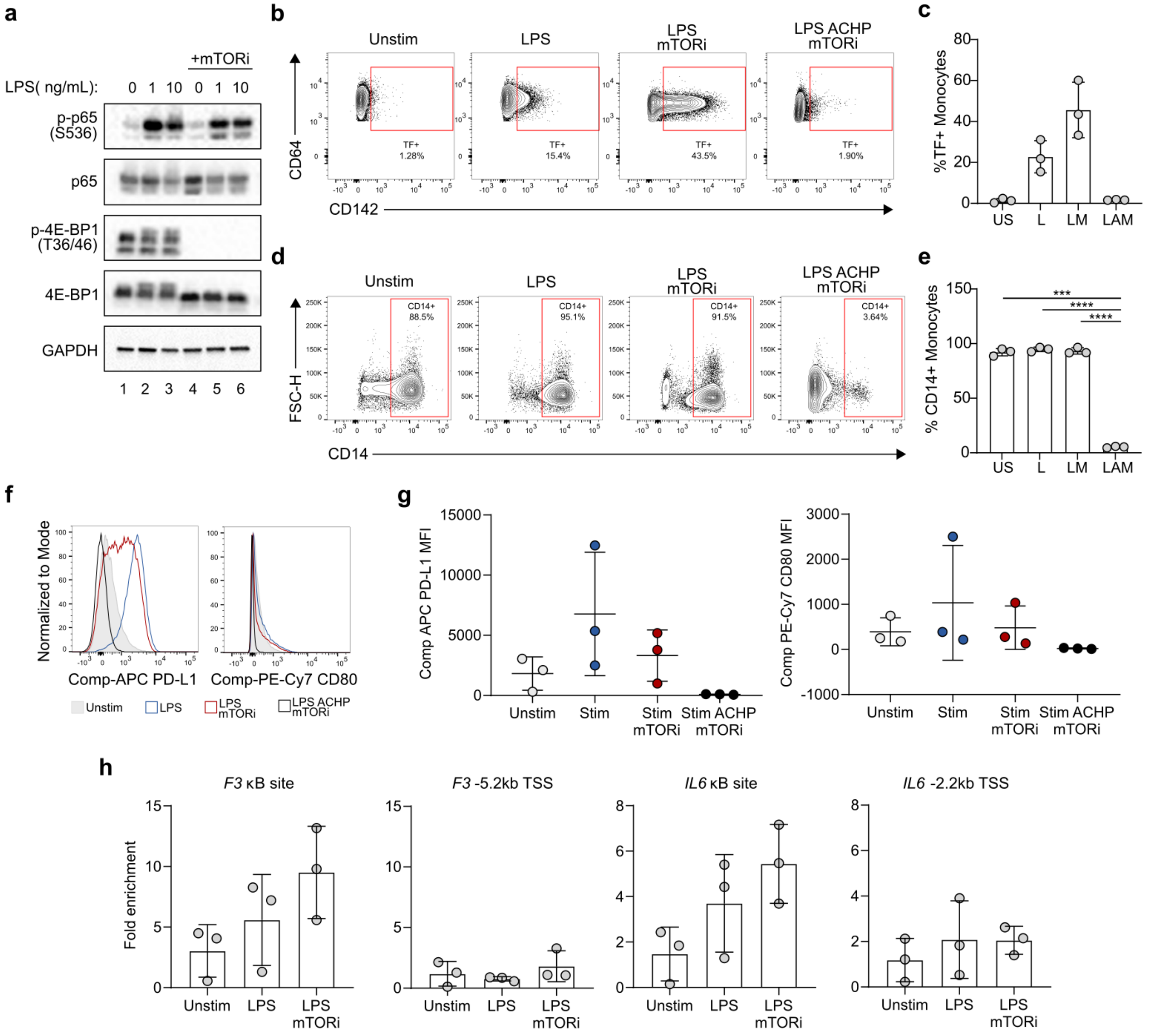

Supplementary Fig 2. Potentiation of surface tissue factor expression by mTORi pretreatment of LPS-stimulated monocytes is NF-κB-dependent. A Monocytes from one donor were pretreated with an mTORi (AZD2014, 5 μM) or DMSO for 6 hrs prior to brief stimulation with LPS at the indicated concentration (0-10 ng/mL) for 30 min, collection, and immunoblot analysis of phosphorylation of p65 and mTOR target 4E-BP1. B-F PBMCs from three independent donors were pretreated with an mTORi (AZD2014, 5 μM) and an inhibitor of IκB kinase (ACHP, 5 μM), mTORi alone, or DMSO, and stimulated with LPS (1ng, 12h) prior to staining for flow cytometry, staining for TF/CD142 (B, C), CD14 (D, E), PD-L1 (F, G), and CD80 (F, G). F Representative plots show unstimulated monocytes (gray, filled), LPS-stimulated only (blue), LPS-stimulated with mTORi pretreatment (red), and with ACHP treatment (black). Given the sensitivity of CD14 to ACHP treatment, monocyte populations are defined in these flow plots by leukocyte/singlet/live/CD64^{+}. H Monocytes were pretreated with an mTORi (AZD2014 at 5 μM, 6h) or DMSO and stimulated with LPS (1 ng, 6h) for directed chromatin immunoprecipitation (ChIP-PCR), amplifying from two loci (*F3* and *IL6*) using primers targeting κB site proximal to the TSS and an upstream DNase I-insensitive region (negative control). Fold change of enrichment with p65 relative to mock IgG control shown. ChIP analysis represents

data from three independent donors, and technical duplicates for both IP and real-time qPCR. Signficance was determined via one-way ANOVA and Tukey's multiple comparisons, except in cases where Friedman tests and Dunn's multiple comparisons were appropriate..***$p<0.001$, ****$p<0.0001$. Error bars represent mean+/-SD.

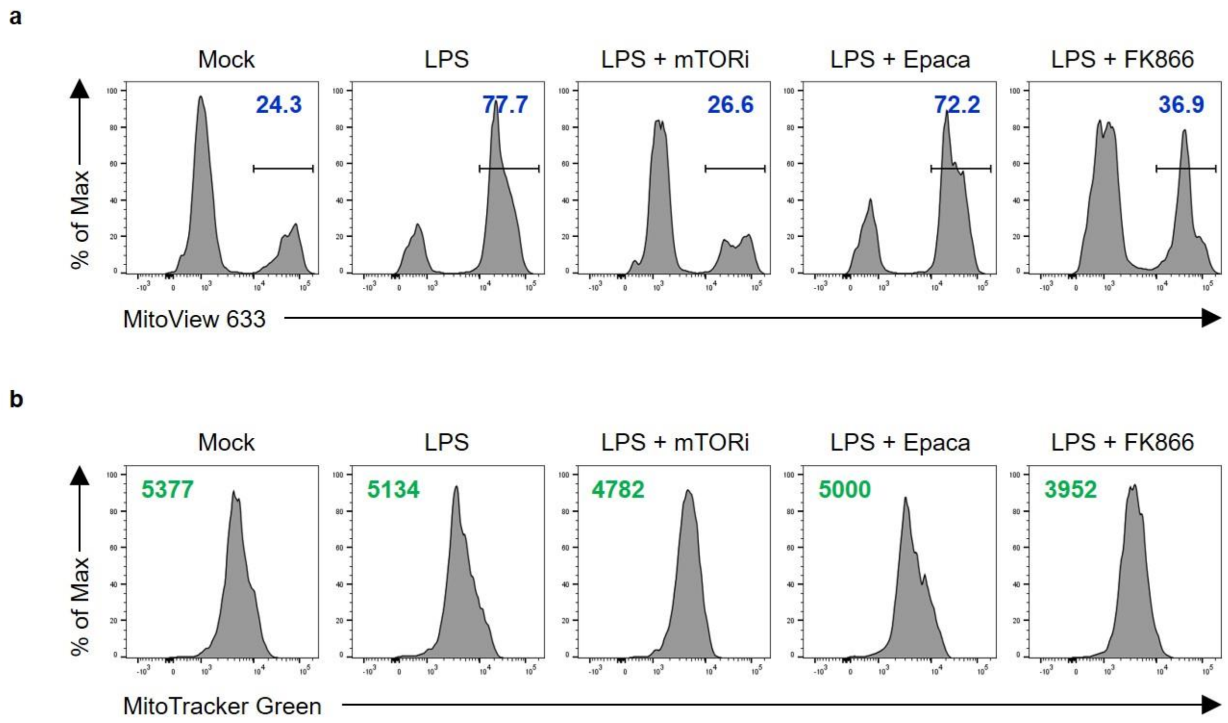

Supplementary Figure 3. Analysis of mitochondrial membrane potential (ΔΨm) and mass in mTORi-treated monocytes. Representative histogram plots from primary monocytes pretreated for 6 h with indicated inhibitors and stimulated with LPS (1 ng, 18 h) prior staining with MitoView 633 (a) and MitoTracker Green (b). (a) The percentage of gated cells with high ΔΨm are shown in blue, while the mitochondrial mass of treated cells (b), as measured by mean flourenscence intensity of MTG are shown in green. mTori is AZD2014 (5 μM). IDO1 inhibitor is epacadostat (1 μM). NAMPT inhibitor is FK866 (100 nM).

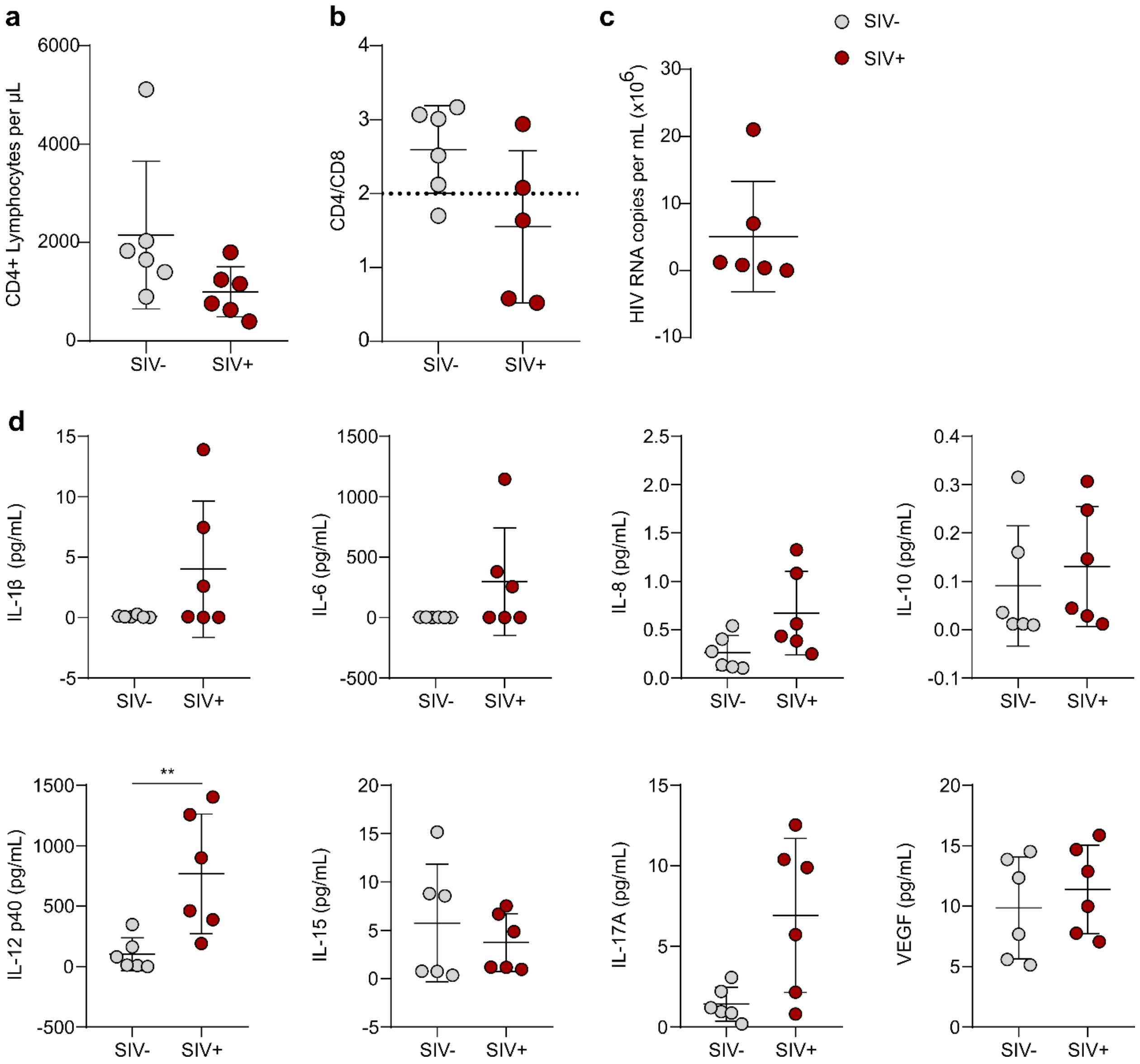

Supplementary Fig 4. Markers of disease progression among study animals. A-C CD4^{+} T cell counts, CD4/CD8 ratio, and results of viral load testing of study animals on the date of PBMC cryopreservation. D MSD analysis of plasma from uninfected (gray) and SIVmac239-infected rhesus macaques (red) at the same time point as in A-C. For cytokine analysis, a Mann-Whitney *U* test was used, **p<0.01, two-tailed. Error bars represent mean+/-SD.

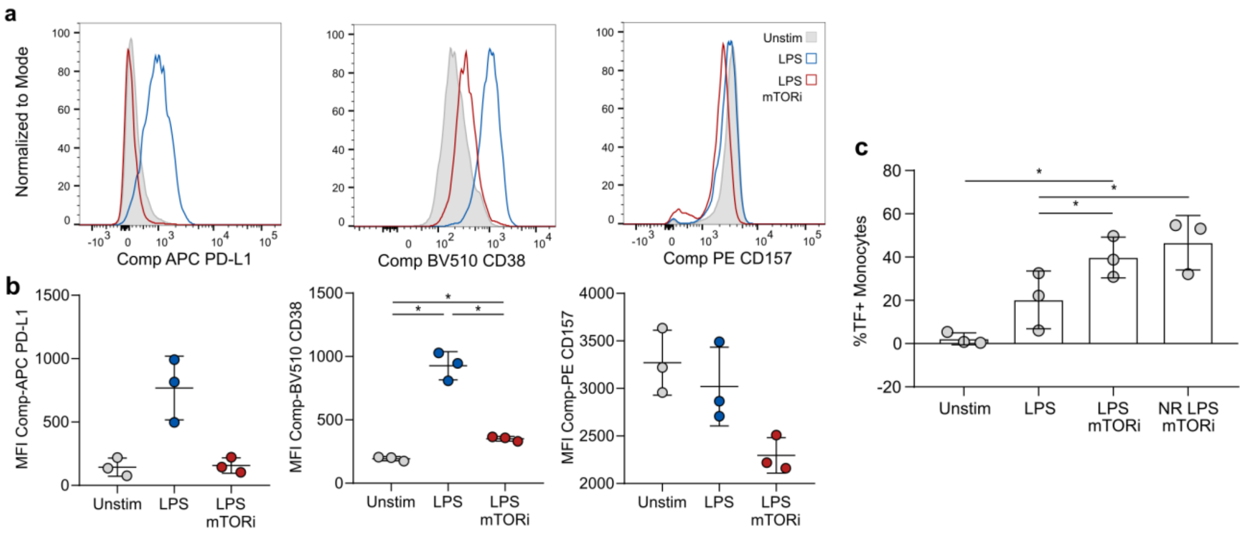

Supplementary Fig 5. Differential surface expression of NAD$^+$ hydrolases on human primary monocytes. A-B PBMCs from three independent donors were pretreated with mTORi (AZD2014 5 μM) or DMSO overnight prior to stimulation with LPS (1ng, 24h). Representative plots (A) show unstimulated monocytes (gray, filled), LPS-stimulated only (red), and LPS-stimulated with mTORi pretreatment (blue). C PBMCs from three independent donors were pretreated with an mTORi (AZD2014 5 μM) in media supplemented with or without NR (100 μM), or DMSO, for 6h prior to stimulation with LPS (1 ng, 12h) and staining for TF, assessed via flow cytometry. Flow data aggregates (B and C) represent gating on leukocyte/singlet/live/CD64$^+$/CD14$^+$. Significance was calculated via one-way ANOVA and Tukey's multiple comparisons. *$p<0.05$. Error bars represent mean+/-SD.

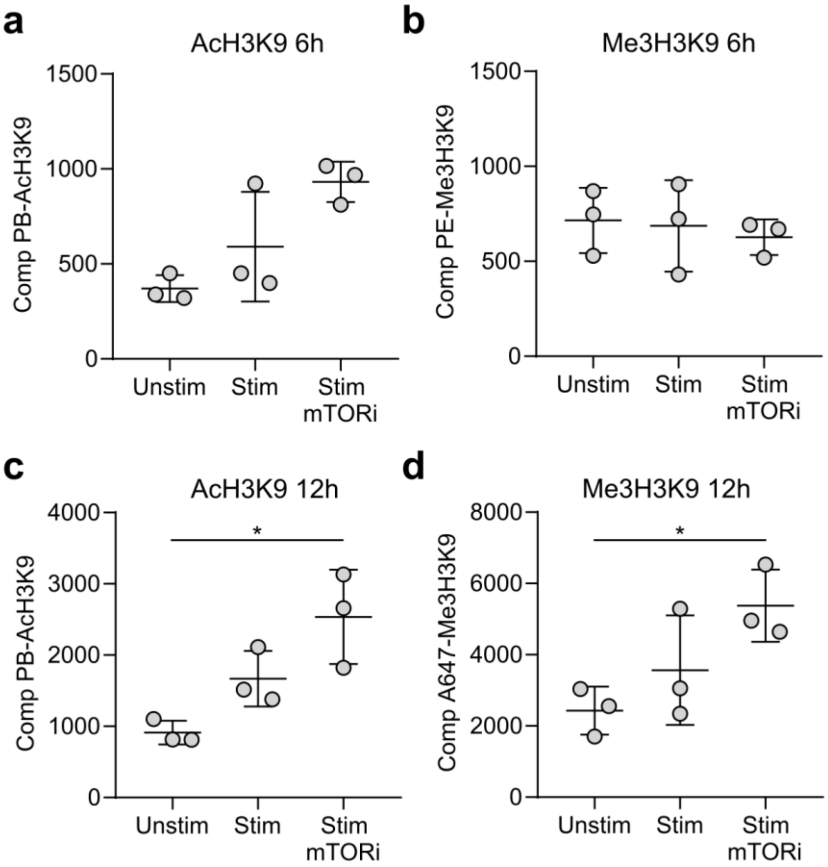

Supplementary Fig 6. mTOR inhibition promotes global accumulation of sirtuin target acH3K9. A-B PBMCs from three independent donors were pretreated with an mTORi (AZD2014 at 5 μM, 6h) or DMSO and stimulated with LPS (1 ng, 6 h) prior to intracellular staining for global acetylated (A) or trimethylation (B) at H3K9. C-D PBMCs from three additional donors were pretreated with an mTORi (AZD2014 at 5 μM, 6h) or DMSO and stimulated with LPS (1 ng, 12 h) prior to intracellular staining for global acetylated (A) or trimethylation (B) at H3K9. Flow data aggregates represent gating on leukocyte/singlet/live/$CD64^{+}$/CD14+. Significance was determined via one-way ANOVA and Tukey's multiple comparisons, except (C), for which a nonparametric test of variance and Dunn's multiple comparisons were used. *p<0.05. Error bars represent mean+/-SD.

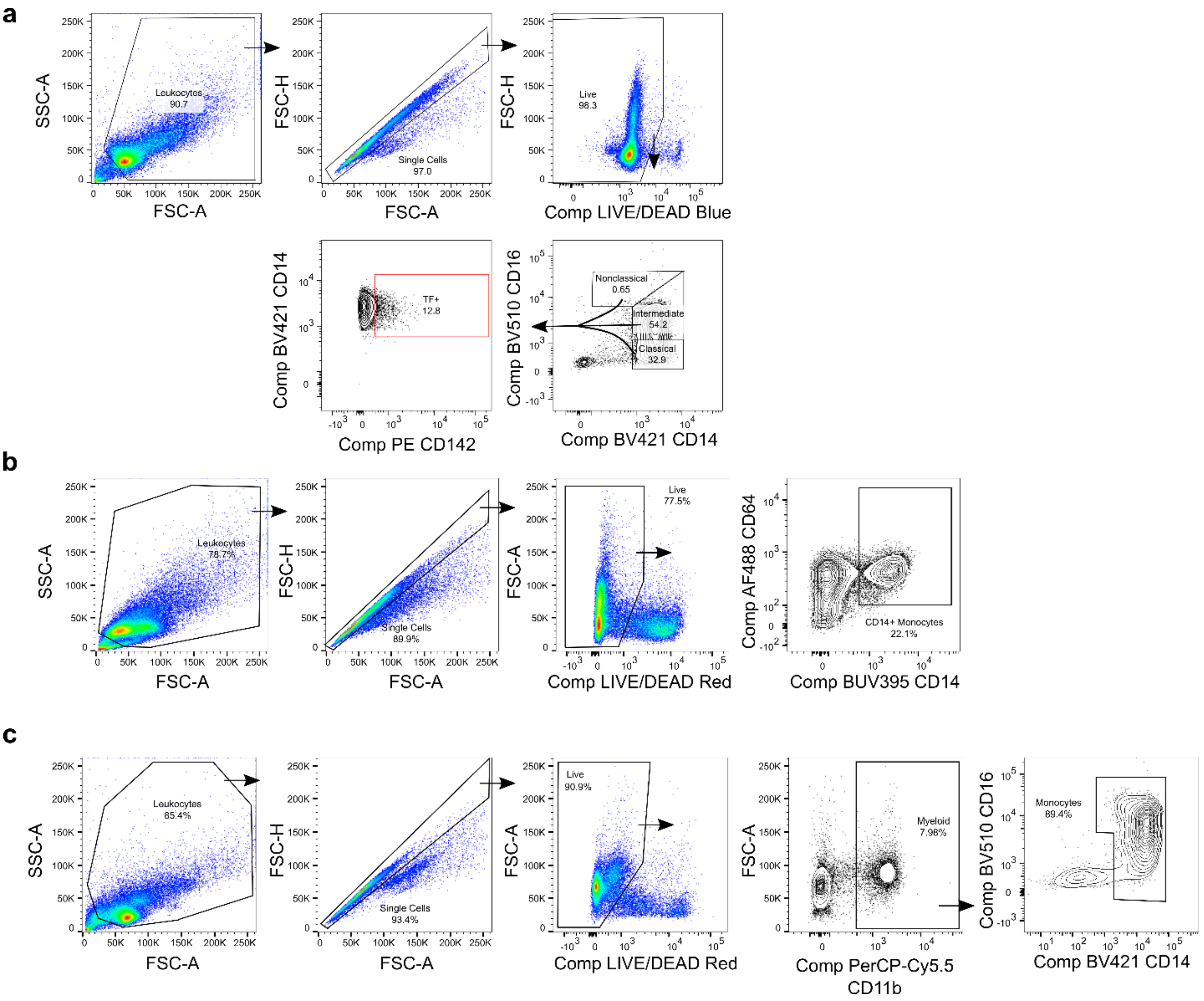

Supplementary Fig 7. Gating strategies. A Gating strategy for human specimens based on traditional monocyte subtypes. B Gating strategy for human specimens employing CD64. C Gating strategy used for NHP specimens.

Supplementary Table 1. Study animal characteristics

Animal ID	Sex	Age (y)	Disease Status	DPI	Virus	VL (x10^6)	CD4 Count
RHDFTF	M	7	Uninfected	NA	NA	NA	901.18
RHDG3H	M	4	Uninfected	NA	NA	NA	1402.674
RHDGNH	M	4	Uninfected	NA	NA	NA	1830.976
RHDGNW	M	4	Uninfected	NA	NA	NA	1653.012
RHDGRA	M	4	Uninfected	NA	NA	NA	2032.569
RHDGTP	M	4	Uninfected	NA	NA	NA	5107.77
RH37033	M	14	Chronic	600	SIVmac239	0.38	1246.57
RH37034	M	14	Chronic	600	SIVmac239	0.0065	635.58
RHDFH4	M	6	Chronic	350	SIVmac239	1.2	400.14
RHDFiV	M	8	Chronic	350	SIVmac239	0.81	766.584
RHDG2V	M	4	Chronic	240	SIVmac239	21	1159.131
RHDGME	M	5	Chronic	210	SIVmac239	7.0	1803.445

Supplementary Table 2. Conjugated antibodies

Target	Clone	Manufacturer
CD3*	SP34-2	BD
CD4*	OKT4	BioLegend
CD8*	RPA-T8	BD
CD11b*	M1/70	BioLegend
CD14	MTP9	BD
CD14*	M5E2	BD
CD16*	3G8	BD
CD38	HIT2	Biolegend
CD64	10.1	BD
CD80	L307.4	BD
CD157	SY/11B5	BD
PD-L1	B7-H1	BD
TF*	HTF-1	Thermo/eBioscience
IL-1β	JK1B-1	BioLegend
IL-6	MQ2-13A5	Thermo/eBioscience
TNF-α	MAb11	BioLegend

*Used in staining of NHP PBMC

Supplementary Table 3. Unconjugated antibodies

Target	Clone	Manufacturer
P-4E-BP1 (Thr37/46)	236B4	Cell Signaling Technologies
4E-BP1	53H11	Cell Signaling Technologies
P-S6 (Ser235/236)	D57.2.2E	Cell Signaling Technologies
S6	5G10	Cell Signaling Technologies
GAPDH	GAPDH-71.1	Millipore Sigma
GAPDH	14C10	Cell Signaling Technologies
P-p65 (Ser536)	93H1	Cell Signaling Technologies
p65	D14E12	Cell Signaling Technologies
NAMPT	BLR058F	Bethyl Laboratories
IDO	D5J4E	Cell Signaling Technologies
p65	n/a	Abcam (ab16502)

Supplementary Table 4. Conventional ChIP primer sequences

Target	Forward (5' to 3')	Reverse (5' to 3')
F3 kB site	CCAGAGCCCGTGCTTTCTAT	TCACCCCAGTGATTCAACCG
F3 negative (-5.2kB)	CCCCTTATGGCAAGGAATAGAA	GCCTTTGATGGTGATGCTCC
IL6 kB site	CGCTAGCCTCAATGACGACC	TGGGGCTGATTGGAAACCTT
IL6 negative (-2.2kB)	GCAAGACGCAAGCTGGACTA	CAGCTCGGCTATATCGGTTCA
