# Supplementary material for: mTOR regulation of metabolism limits LPS-induced monocyte inflammatory and procoagulant responses
Source: Commun Biol. 2022 Aug 26;5:878. doi: 10.1038/s42003-022-03804-z (PMC9412771; doi:10.1038/s42003-022-03804-z)
Supplement: Supplementary file 2 — Description of Additional Supplementary Files [file 42003_2022_3804_MOESM2_ESM.pdf]

mTOR regulation of metabolism limits LPS-induced monocyte inflammatory and procoagulant responses

DESCRIPTION OF SUPPLEMENTARY FILES

Supplementary Data 1

Metabolomic data set corresponding to Figure 4a-c. Naming of samples follows the convention of Donor_Treatment. Treatment numbers refer to (1) DMSO-pretreated/unstimulated, (2) DMSO-pretreated/LPS-stimulated, and (3) mTORi-pretreated/LPS-stimulated. Values represent relative peak values prior to normalization.

Supplementary Data 2

Metabolomic data set corresponding to Figure 4g-h. Naming of samples follows the convention of Sample_Donor_Treatment. Treatment letters refer to (A) DMSO-pretreated/unstimulated, (B) DMSO-pretreated/LPS-stimulated, and (C) mTORi-pretreated/LPS-stimulated. Values represent relative peak values prior to normalization.
