# Supplementary material for: mTOR regulation of metabolism limits LPS-induced monocyte inflammatory and procoagulant responses
Source: Commun Biol. 2022 Aug 26;5:878. doi: 10.1038/s42003-022-03804-z (PMC9412771; doi:10.1038/s42003-022-03804-z)
Supplement: Supplementary file 5 — Reporting Summary [file 42003_2022_3804_MOESM5_ESM.pdf]

nature research

Corresponding author(s): Harry E. Taylor, PhD and Richard T. D'Aquila, MD
DBPR COMMSBIO-20-3523A

Last updated by author(s): August 1, 2022

# Reporting Summary

Nature Research wishes to improve the reproducibility of the work that we publish. This form provides structure for consistency and transparency in reporting. For further information on Nature Research policies, see our Editorial Policies and the Editorial Policy Checklist.

## Statistics

For all statistical analyses, confirm that the following items are present in the figure legend, table legend, main text, or Methods section.

| n/a | Confirmed | |
|---|---|---|
| ☐ | ☒ | The exact sample size ($n$) for each experimental group/condition, given as a discrete number and unit of measurement |
| ☐ | ☒ | A statement on whether measurements were taken from distinct samples or whether the same sample was measured repeatedly |
| ☐ | ☒ | The statistical test(s) used AND whether they are one- or two-sided<br>*Only common tests should be described solely by name; describe more complex techniques in the Methods section.* |
| ☒ | ☐ | A description of all covariates tested |
| ☐ | ☒ | A description of any assumptions or corrections, such as tests of normality and adjustment for multiple comparisons |
| ☐ | ☒ | A full description of the statistical parameters including central tendency (e.g. means) or other basic estimates (e.g. regression coefficient) AND variation (e.g. standard deviation) or associated estimates of uncertainty (e.g. confidence intervals) |
| ☐ | ☒ | For null hypothesis testing, the test statistic (e.g. $F$, $t$, $r$) with confidence intervals, effect sizes, degrees of freedom and $P$ value noted<br>*Give P values as exact values whenever suitable.* |
| ☒ | ☐ | For Bayesian analysis, information on the choice of priors and Markov chain Monte Carlo settings |
| ☒ | ☐ | For hierarchical and complex designs, identification of the appropriate level for tests and full reporting of outcomes |
| ☒ | ☐ | Estimates of effect sizes (e.g. Cohen's $d$, Pearson's $r$), indicating how they were calculated |

*Our web collection on statistics for biologists contains articles on many of the points above.*

## Software and code

Policy information about availability of computer code

| | |
|---|---|
| Data collection | BD FACSDiva v8.0.1 |
| Data analysis | GraphPad Prism v8.0.1, R v3.6.1 |

For manuscripts utilizing custom algorithms or software that are central to the research but not yet described in published literature, software must be made available to editors and reviewers. We strongly encourage code deposition in a community repository (e.g. GitHub). See the Nature Research guidelines for submitting code & software for further information.

## Data

Policy information about availability of data

All manuscripts must include a data availability statement. This statement should provide the following information, where applicable:

- Accession codes, unique identifiers, or web links for publicly available datasets
- A list of figures that have associated raw data
- A description of any restrictions on data availability

Figures 3 and 4 have associated raw data. These data, and others, that support this study are available from the corresponding authors on reasonable request. RNA-seq data has been uploaded to the Gene Expression Omnibus (accession GSE187403) and will be made publicly available following publication.

# Field-specific reporting

Please select the one below that is the best fit for your research. If you are not sure, read the appropriate sections before making your selection.

☒ Life sciences ☐ Behavioural & social sciences ☐ Ecological, evolutionary & environmental sciences

For a reference copy of the document with all sections, see nature.com/documents/nr-reporting-summary-flat.pdf

# Life sciences study design

All studies must disclose on these points even when the disclosure is negative.

| | |
|---|---|
| Sample size | Specimens from six different human subjects were used for the RNAseq experiment based on the advice of the experienced core providing this service (NUSeq). |
| Data exclusions | Prior to statistical analysis, the metabolomic data set was cleaned by removing targets with less than 66% non-zero values and outliers that were detected using a random forest based method. |
| Replication | For experiments involving human blood cells purchased from a blood bank and shipped overnight, the cell conditions on arrival were rarely not perfect. Adequate cell quality was confirmed by using only cells from donors that responded to LPS stimulation to assure reproducibility. |
| Randomization | No randomization was used for the purposes of this study. Ex vivo treatments were matched among donors. |
| Blinding | None of the measures used required scoring methods highly susceptible to bias. |

# Reporting for specific materials, systems and methods

We require information from authors about some types of materials, experimental systems and methods used in many studies. Here, indicate whether each material, system or method listed is relevant to your study. If you are not sure if a list item applies to your research, read the appropriate section before selecting a response.

## Materials & experimental systems

| n/a | Involved in the study | |
|---|---|---|
| ☐ | ☒ | Antibodies |
| ☒ | ☐ | Eukaryotic cell lines |
| ☒ | ☐ | Palaeontology and archaeology |
| ☐ | ☒ | Animals and other organisms |
| ☒ | ☐ | Human research participants |
| ☒ | ☐ | Clinical data |
| ☒ | ☐ | Dual use research of concern |

## Methods

| n/a | Involved in the study | |
|---|---|---|
| ☒ | ☐ | ChIP-seq |
| ☐ | ☒ | Flow cytometry |
| ☒ | ☐ | MRI-based neuroimaging |

## Antibodies

| | |
|---|---|
| Antibodies used | See Extended Data Tables 2 and 3 for antibody details. |
| Validation | Antibody uses match species reactivity and applications reported by each manufacturer.<br><br>BioLegend is committed to supporting research by providing high quality, validated antibodies. Cell Signaling Technologies antibodies are validated by the manufacturer for stated application using human specimens. Millipore Sigma cites application of anti-GAPDH antibody in Burke, J.R., et al., Nature Med. 2,347-350 (1996), among other sources. Bethyl has validated the anti-NAMPT antibody for WB analysis of human specimens. Thermo/eBioscience antibodies used were pre-titrated and tested by flow cytometric analysis of stimulated normal human peripheral blood cells by the manufacturer. BD conducts rigorous QC testing in primary model systems and of each antibody lot tested side-by-side with a previously produced lot as reference. Abcam ab16502 is KO validated by the manufacturer. |

## Animals and other organisms

Policy information about studies involving animals; ARRIVE guidelines recommended for reporting animal research

| | |
|---|---|
| Laboratory animals | See Extended Data Table 1 for study animal details. |
| Wild animals | The study did not involve wild animals. |
| Field-collected samples | The study did not involve samples collected from the field. |

| | |
|---|---|
| Ethics oversight | Animals were housed and cared in accordance with American Association for Accreditation of Laboratory Animal Care standards in AAALAC accredited facilities, and all animal procedures were performed according to protocols approved by the Institutional Animal Care and Use Committees of the National Institute of Allergy and Infectious Diseases under animal study protocol LVD26. |

Note that full information on the approval of the study protocol must also be provided in the manuscript.

## Flow Cytometry

### Plots

Confirm that:

☒ The axis labels state the marker and fluorochrome used (e.g. CD4-FITC).

☒ The axis scales are clearly visible. Include numbers along axes only for bottom left plot of group (a 'group' is an analysis of identical markers).

☒ All plots are contour plots with outliers or pseudocolor plots.

☒ A numerical value for number of cells or percentage (with statistics) is provided.

### Methodology

| | |
|---|---|
| Sample preparation | Peripheral blood mononuclear cells (PBMC) were isolated via density gradient centrifugation using Ficoll-Paque PLUS (GE Healthcare, Pittsburg, PA) from uninfected donors' EDTA-coagulated leukopacks (Lifesource, Rosemont, IL and New York Blood Center, New York, NY). Monocytes were purified through magnetic-assisted cell sorting (Pan-Monocyte Isolation Kit, Miltenyi Biotec, Bergisch Gladbach, Germany) and resuspended in complete RPMI plus 10% defined FBS (GE Healthcare) and penicillin/streptomycin.<br><br>Following recovery from culture, PBMCs or monocytes were washed once in ice cold PBS prior to staining for flow. |
| Instrument | BD LSRII Fortessa SORP Cell Analyzer |
| Software | BD FACS Diva v8.0.1 |
| Cell population abundance | No cell sorting was performed |
| Gating strategy | Dead cells and debris were excluded by gating on the whole leukocyte population seen on FSC/SSC. Singlets were gated by FSC-H/FSC-A. Dead cells were excluded using LIVE/DEAD cell stain (Invitrogen). For more details on gating of monocyte populations, see Extended Data Figure 6. Gating of TF+ populations was determined by using FMO controls. |

☒ Tick this box to confirm that a figure exemplifying the gating strategy is provided in the Supplementary Information.
